# Rapid identification of chrysanthemum teas by computer vision and deep learning

**DOI:** 10.1002/fsn3.1484

**Published:** 2020-03-03

**Authors:** Chunlin Liu, Weiying Lu, Boyan Gao, Hanae Kimura, Yanfang Li, Jing Wang

**Affiliations:** ^1^ Beijing Advanced Innovation Center for Food Nutrition and Human Health Beijing Technology & Business University (BTBU) Beijing China; ^2^ Institute of Food and Nutraceutical Science School of Agriculture and Biology Shanghai Jiao Tong University Shanghai China

**Keywords:** chrysanthemum tea, computer vision classification, deep neural network, morphological feature

## Abstract

Seven commercial Chinese chrysanthemum tea products were classified by computer vision combined with machine learning algorithms. Without the need of building any specific hardware, the image acquisition was achieved in two computer vision approaches. In the first approach, a series of multivariate classification models were built after morphological feature extraction of the image. The best prediction accuracies when classifying flowering stages and tea types were respectively 90% and 63%. In comparison, the deep neural network was applied directly on the raw image, yielded 96% and 89% correct identifications when classifying flowering stage and tea type, respectively. The model can be applied for rapid and automatic quality determination of teas and other related foods. The result indicated that computer vision, especially when combined with deep learning or other machine learning techniques can be a convenient and versatile method in the evaluation of food quality.

## Graphical abstract

This manuscript reported the multivariate classification of seven commercial Chinese chrysanthemum tea products by computer vision combined with machine learning algorithms. The result indicated that computer vision, especially when combined with deep learning or other machine learning techniques can be a convenient and versatile method in the evaluation of food quality.

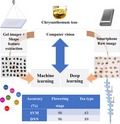

## 1 INTRODUCTION

The chrysanthemums are the flowering perennial plants with enormous horticultural varieties and cultivars. They are widely used as a raw ingredient of functional food products in China and other East Asian countries, because they have ornamental value as well as functional benefits after consumption. As a healthy functional food, some chrysanthemum plants such as *Chrysanthemum morifolium* Ramat. and *Coreopsis tinctoria* are traditionally consumed as teas. Chrysanthemum teas are dried capitate inflorescence of the chrysanthemums, and usually consumed in combination with hot or boiling water. They are considered to have many beneficial effects, most typically are anti‐inflammation (Li, Yang, et al., 2019; Zhang, Shi, Zhao, Chai, & Tu, 2013) and antioxidant (Li, Yang, et al., 2019; Wang, Xi, Guo, Wang, & Shen, 2015; Yang et al., 2011) and have been applied in treating a series of symptoms such as blurred vision, dermatitis, and eye itch for more than 2,000 years. In our previous studies, it is indicated that different chrysanthemums have different phytochemical compositions together with different health functions (Li, Hao, et al., 2019; Li, Yang, et al., 2019). Therefore, differentiation of chrysanthemum teas can be useful in the quality assurance of its related products.

It is generally accepted that the cultivar, growing environment, and cultivation technique correlates to the chemical compositions, biological properties and final product quality of a botanical (Lu, Jiang, et al., 2014; Lu, Gao, Chen, Charles, & Yu, 2014). A unique cultivation characteristic of chrysanthemum tea products involves in their harvest time. Two different types of chrysanthemum tea products, that is, the fetal and the common chrysanthemum teas, were marketed according to different harvest time. The fetal and common chrysanthemum teas are respectively collected before and in full bloom. Variations in compositional, quality and clinical efficacy existed between the two types of chrysanthemum teas according to a series of researches (Tou, Sun, Zhang, Si, & Liu, 2013; Yuan et al., 2015; Zhou, Yu, Ren, & Wang, 2009). For instance, Zhou et al. demonstrated that the harvest time influences the content of chlorogenic acid in chrysanthemum tea products and suggested that the fetal chrysanthemum harvested before mid‐November yielded better quality (Zhou et al., 2009). In addition, the mineral contents also vary with different flowering stages (Tou et al., 2013). The research of Yuan et al. implied that the fetal chrysanthemum possesses better functional effects than a common chrysanthemum tea “Xiaobaiju” (Yuan et al., 2015). On the other hand, fetal chrysanthemum teas are usually smaller in size compared with common chrysanthemum teas due to different extent of pedal stretching. Furthermore, other cultivation characteristics such as the geographical origination and species were closely related to quality and may also influence the size and shape of teas. Mislabeling and improper use of teas harms the consumers' and producers' mutual interest. Therefore, to better evaluate the quality and promote the utility of chrysanthemums, the classification and quality assessment of chrysanthemum teas are needed.

The quality assessment of chrysanthemum teas was usually performed by instrumental analysis techniques, such as by the gas chromatography–mass spectrometry and olfactometry (Luo, Chen, Gao, Liu, & Wu, 2017). Although precise chemical information can be obtained through this approach, it may not be suitable for rapid assay and high‐throughput, on‐line monitoring due to its relative high cost and long sample pretreatment and analysis time. A computer vision system (CVS) that mimics human vision may be an attractive alternative. By a specially designed image‐recording device combined with a series of image processing methods, CVS not only acquire and process visual information but also make intelligent decisions without any human intervention. The CVS has been already widely used in massive product quality inspection and grading, where repeated and monotonous processing of visual information is needed (Vithu & Moses, 2016). Compared with manual operation, it can achieve a fast, reliable and nondestructive analysis. In addition, with the help of artificial intelligence, automated decisions can be made with complex but mathematically assured models, such that objective, accurate, convenient, and rapid quality detections and identifications can be achieved. Such approach is suitable for large‐scale, on‐line, or at‐line manufacturing of food products.

The CVS is widely applied as a quality assurance technique for food products nowadays. More specifically, the CVS has been extensively studied over the decades in rapidly examining a series of interior and exterior quality metrics such as the varieties, defects and maturities of fruits in grapes (Xia, Wu, Nie, & He, 2016), bananas (Mendoza & Aguilera, 2004), watermelons (Koc, 2007), and rapeseeds (Kurtulmus & Unal, 2015). It is foreseeable that the CVS can be applied in larger fields of industrial applications. However, due to that there is intensive shape deformation during food processing such as the drying process, it is also interesting to examine whether the CVS could predict the quality of food products such as dried foods.

The deep learning is one of the significant advancements in the field of machine learning algorithms in recent years (LeCun, Bengio, & Hinton, 2015). Inspired by the structure of visual cortex, the deep neural network (DNN) is a successful example of complex artificial neural networks that typically bring high classification accuracy on the unstructured data such as image data. Contrary to the previous procedures with the calculation of a shape, color, and texture data set as intermediate feature set for model training, the DNN introduces the concept of convolution core and pool layer, and implements the classification through series of layered network using the entire picture as input. Due that a relatively large‐scaled neural network is applied to simulate human decision process, DNN has already achieved promising results over a wide range of applications involving complicated tasks, such as image classification and speech recognition. Recently, the application of DNN has also been reported in the food industry, such as to evaluate the quality of the fresh‐cut lettuce (Cavallo, Cefola, Pace, Logrieco, & Attolico, 2018), dry‐cured ham slices (Muñoz, Gou, & Fulladosa, 2019), salmon fillets (Xu & Sun, 2018), and the commercially prepared pureed food (Pfisterer, Amelard, Chung, & Wong, 2018).

In this study, two rapid identification approaches for chrysanthemum tea quality were evaluated. The first approach utilized a regular gel imager as hardware workbench, in association with morphological feature extraction and multivariate classification. The second approach applied DNN to the raw image directly. The resulted model was aimed to achieve the automatic discrimination of different types and characteristics of chrysanthemum teas. This approach may help to understand the relationships between appearance and functional components of chrysanthemum teas in the future.

## 2 MATERIALS AND METHODS

### 2.1 Sample collection

Seven representative commercial teas including Hangbaiju (HB), Hangtaiju (HT), Huangshangongju (HG), Kunlunxueju (KX), Kunlunmiju (KM), Huaiju (HJ), and Dabieshantaiju (DT) were collected from different locations in China. The sources and quantities of chrysanthemums used in the experiment were shown in Table 1. The samples were originated in ZheJiang, AnHui, HeNan, and HuBei provinces and Xinjiang Uygur Autonomous Region. These samples were completely dried flowers sealed in plastic bags and stored at ambient temperature. All samples were tested without further processing. In this study, each individual flower bud was treated as an independent instance from the sample. A total of 2,343 and 1,581 instances were collected for shape feature and DNN modeling, respectively.

**Table 1:** Sample information of chrysanthemum teas

ID	Name and Abbreviation	Species	Source	Flowering Stage	Buds Quantity	
					Shape Factors	DNN
1	Hangbaiju (HB)	*Chrysanthemum morifolium* a	Hangzhou, ZheJiang Province	Bloom	203	148
2	Huangshangongju (HG)	*C. morifolium*	Quanjiao, AnHui Province	Bloom	144	97
3	Kunlunxueju (KX)	*Coreopsis tinctoria*	Urumqi, Xinjiang Uygur Autonomous Region	Bloom	332	185
4	Hangtaiju (HT)	*C. morifolium*	Hangzhou, ZheJiang Province	Fetal	348	258
5	Kunlunmiju (KM)	*C. tinctoria*	Urumqi, Xinjiang Uygur Autonomous Region	Fetal	517	373
6	Huaiju (HJ)	*C. morifolium*	Wen County, HeNan Province	Fetal	435	270
7	Dabieshantaiju (DT)	*C morifolium*	HuBei Province	Fetal	364	250

a *C. morifolium*, *Chrysanthemum morifolium* Romat.

### 2.2 Instrumentation

**Figure 1:**
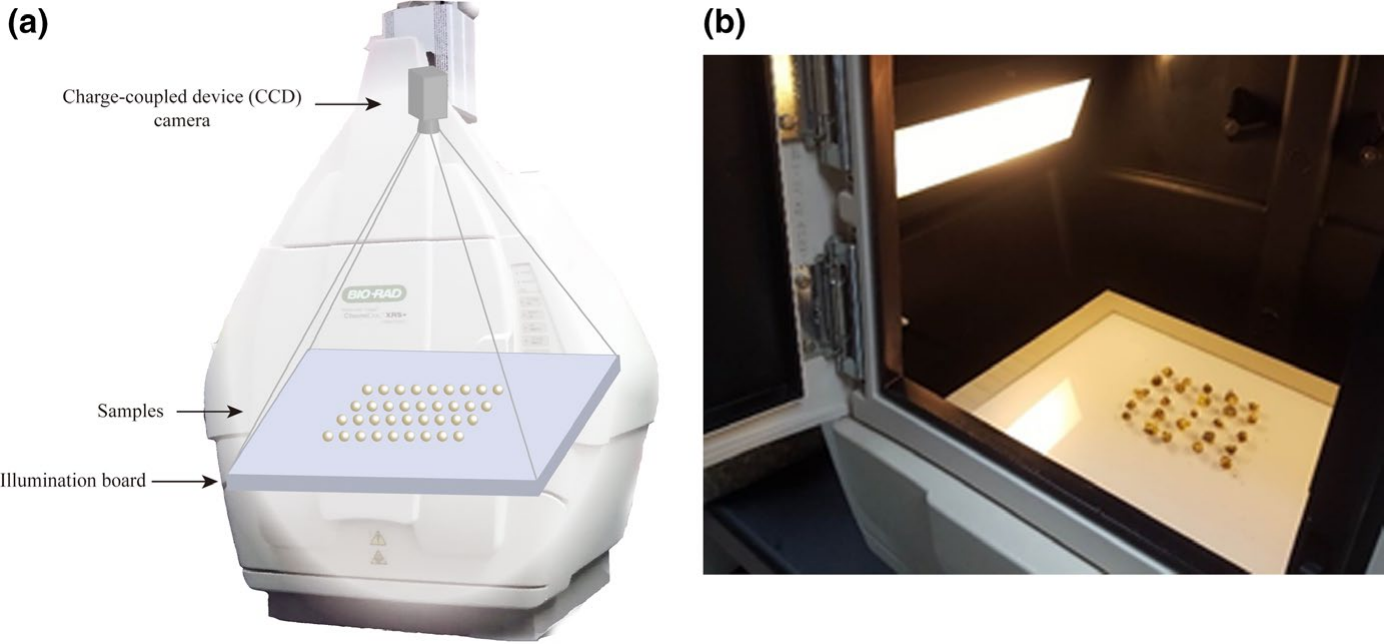
Diagram of image acquisition using a gel imager (a) and arrangement of flower buds (b)

The monochrome contour images were collected from a gel imager. The gel imager consists of a camera, its accompanying illumination devices, a computer and the corresponding software. The camera and illumination devices were applied to capture an image for each sample. A ChemiDoc XRS + gel imager (Bio‐Rad Laboratories) was used as the integrated instrument for camera and illumination in the CVS. The gel imager is a popular instrument used in molecular biology experiments to take snapshots of the gel after electrophoresis. Similar to other specialized CVS hardware, the gel imager is equipped with a high‐resolution, high‐sensitivity charge‐coupled device (CCD) camera as detector. The gel imager was operated at the transmittance mode as demonstrated in Figure 1a, with no additional filter applied. The exposure time was 0.1 s, and light source was white light transmission. A grayscale image was collected in a 12.0 × 9.0 cm rectangle area with resolutions at 1,392 × 1,040 pixels during each run. The horizontal resolution and the vertical resolution were both 295 dpi, with 16 bit‐depth. All images were stored in the Tagged Image File Format (TIFF). The acquisition was in Coomassie blue mode for an ordinary protein gel shot. Typically, 16–25 samples were included in each shot in an organized order (Figure 1b). The data were collected using the ImageLab software (version 4.1, Bio‐Rad Laboratories).

The color images were collected by a smartphone. A Huawei Mate 10 smartphone (model ALP‐AL00, Huawei Technologies Co., Ltd) is operated manually without any mounting device. The focal length was 4 mm, and the focal stop was f/1.6. The ISO speed was ISO‐50. No flash was applied. The resulted color image was in the Joint Photographic Experts Group (JPEG) format with a 3,968 × 2,976 resolution with a 24 bit‐depth. The compression rate was 0.95 compressed bits/pixel. The horizontal resolution and the vertical resolution were both 96 dpi. All samples were placed on an ordinary A4 printing paper (210 × 297 mm) printed with four finder patterns. The patterns were similar to the quick response (QR) code to locate the edges of the 10 cm × 10 cm sample area for perspective distortion. The perspective correction of the images was performed to extract the area. Afterward, the same image segmentation routine was applied to extract the images of individual buds for further DNN classification.

### 2.3 Image processing and data analysis

The routines for CVS image processing include a sequence of functionalities including image pre‐processing, segmentation, contour extraction, and morphological features calculation in a fully automated manner. The image preprocessing was applied to obtain proper contour image of chrysanthemum teas. A median filter with a template of 3 × 3 pixel was used to de‐noise and enhance the image. Afterward, the image was mapped to binarized intensity using the Otsu's method (Otsu, 1979), followed by segmentation to subimages of individual flower buds. The image contour was then computed by the “findContours” function in OpenCV. The morphological features were calculated based on the image contour, and the morphological feature dataset were obtained. Fourteen contour features that describe representative properties of image contour were described in Table 2. Besides the commonly used features such as the perimeter, area, and roundness, three metrics were proposed to measure the irregularity. The irregularity metric was calculated by the variance of contour distances, where the contour distances are defined as the distances between all contour points on the outline and the center of mass. Additionally, the normalized irregularity with a similar calculation procedure to the irregularity feature, except that all the contour distances were divided by the mean contour distance. Similar to roundness, the irregularity metrics also describe the extent to which the object is similar to a circle. When the object is closer to a round shape, the irregularity will be lower, and vice versa. These shape descriptors were compared individually or taken together by chemometrics to study tea quality with specific quantitative models of interest.

**Table 2:** List of morphological features

Feature	Description
Perimeter	Summed Euclidean distances between the consecutive points in the contour
Area	Number of pixels in the shape
Long‐axis length	Distance between the two farthest points on the outline
Short‐axis length	Length of the line that perpendicular to the long axis, and passing through the center of mass
Incircle radius	Radius of the largest incircle of the shape
Excircle radius	Radius of the smallest excircle of the shape
Area equivalent diameter	$\sqrt{4\;Area/\pi }$
Circularity	4π∙Area/Perimeter^2^
Shape parameter	Perimeter^2^/Area
Aspect ratio	Long‐axis length/Short‐axis length
Compactness	Area equivalent diameter/Long‐axis length
Roundness	Incircle radius/Excircle radius
Irregularity	Variance of distances between all contour points on the outline and the center of mass
Normalized irregularity	Variance of normalized Euclidean distances between all contour points on the outline and the center of mass

The morphological feature dataset was projected to a two‐dimensional space and visualized by the principal component analysis (PCA), a widely applied statistical method that transforms the correlated variables into a set of linear uncorrelated variables by orthogonal transformation. The principal component score and loading of each of the tea samples was plotted as the coordinate axis after PCA, in which each sample were displayed as points to achieve a direct classification.

Before the supervised learning, the z‐score standardization (autoscaling) was used. The z‐score standardization implements mean‐subtraction and rescaling of the features, such that all features have means of zero with standard deviations of one. After the transformation, the influences of units of features, as well as abnormal data were removed. Besides unsupervised PCA, chrysanthemum classifications by the supervised learning methods were also tested. The entire data are randomly divided by a 9:1 ratio into a training set (2,112 and 1,423 samples for shape factors and DNN, respectively) and an independent test set (231 and 158 samples for shape factors and DNN, respectively) to establish a classifier for flowering stage and tea type classification.

Three representative machine learning classifiers were evaluated in this study, including the k‐nearest neighbor (KNN), multiple linear perceptron (MLP), and support vector machine (SVM). Because appropriate selection of parameters is the key to achieve optimal performances of the classifier, cross‐validation were applied for all classifiers. Specifically, a grid‐search algorithm by a 10‐fold cross‐validation on solely the training set was used to determine the optimal parameters, and the optimal hyperparameters were selected with the highest prediction accuracy. For the KNN, the number of nearest neighbors (*k*) were searched in the grid as *k* = 1, 2, 3, 4, 5, together with *p* = 2, 3, where p is the power parameter for the Minkowski metric. For the MLP, the training with the following parameters was included in the grid search: hidden layer size = 5,7,9, … 29, with both the rectified linear units (ReLU) and the hyperbolic tangent (tanh) functions were tested as the activation function. The Adam optimization algorithm was selected as the MLP solver. For the SVM, the radial basis function (RBF) was selected as the kernel function. A series of penalties (*C*) and kernel radii (*γ*) was searched in the grid as follows: *C* = 2^0^, 2^1^, 2^2^, …, 2^10^, *γ* = 2^–8^, 2^–7^, 2^–6^, …, 2^0^. All other parameters were kept unchanged.

**Figure 2:**
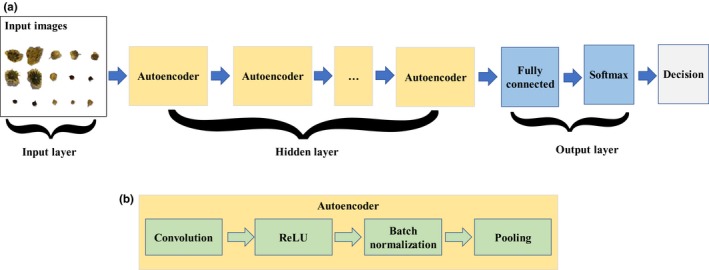
Diagrams of deep convolutional neural network architecture (a) and autoencoder unit (b)

For the DNN, segmented raw images instead of hand‐crafted features were used for the network. Each of the chrysanthemum buds was recognized and extracted, and then, they were downsized to 50 × 50 pixels by bicubic interpolation. The input layer consisted of 7,500 neurons from 50 × 50 image dimension with three color channels of red, green and blue (RGB). The convolutional neural network (CNN) is selected as the DNN model, as it is particularly useful for finding patterns in images to recognize objects. The hidden layers of the DNN consisted of a series of stacked layers organized in repeated units that referred to as autoencoders. Each autoencoder was a combination of a convolution layer, a ReLU layer, a batch normalization layer, and an average pooling layer. Specifically, a convolution layer which performs convolution operation over an image, with the number of parameters to be learned in these filters is equal to the number of elements of these filters; a nonlinear ReLU layer that perform a transformation similar to the sigmoid function are placed right under each of the convolution layers; a batch normalization layer which normalize outputs from the previous layer to speed up training of convolutional neural networks; a pooling layer reduces the size of the image by performing a sliding‐window averaging function to subsample the original image. The final output of the network is passed to a softmax layer. The entire network structure is depicted in Figure 2.

The hyperparameters of the network architectures were optimized by training a series of different architectures as candidates, taking the depth of the network, the sizes of the convolutional filters and pooling windows, and the total number of convolutional filters in the pooling layer together into consideration. The architecture was based on VGG net (Simonyan & Zisserman, 2015), in which the number of convolutional filters generally doubles with the depth of the network. The training parameters were as follows: the optimizer was the root mean square propagation (RMSProp); the learning rate stayed constantly at 1 × 10^–4^; the maximum number of epochs was 100; the minibatch size was 128.

The routines for CVS image processing were developed in‐house using Python (version 3.6, Python Software Foundation, http://www.python.org) with the Open Source Computer Vision Library (OpenCV Library, version 3.4, https://opencv.org/). Python and MATLAB (version 2019a, The MathWorks, Inc) programing languages were used to perform data analyses and classification. The numpy (version 1.15.2) and scikit‐learn machine learning (version 0.20.0) modules for Python, and the deep learning toolbox (version 12.1) for MATLAB were used. All calculation was performed on a personal computer with a Ryzen 5 3600X processor (Advanced Micro Devices) and 32 GB of RAM, running on 64‐bit windows 10 operating system (Microsoft). The DNN calculation was accelerated by a Nvidia GeForce GTX 1,060 graphics card (Nvidia Corporation) with 6 GB memory.

## 3 RESULTS AND DISCUSSION

### 3.1 Comparison of raw images and shape features

**Figure 3:**
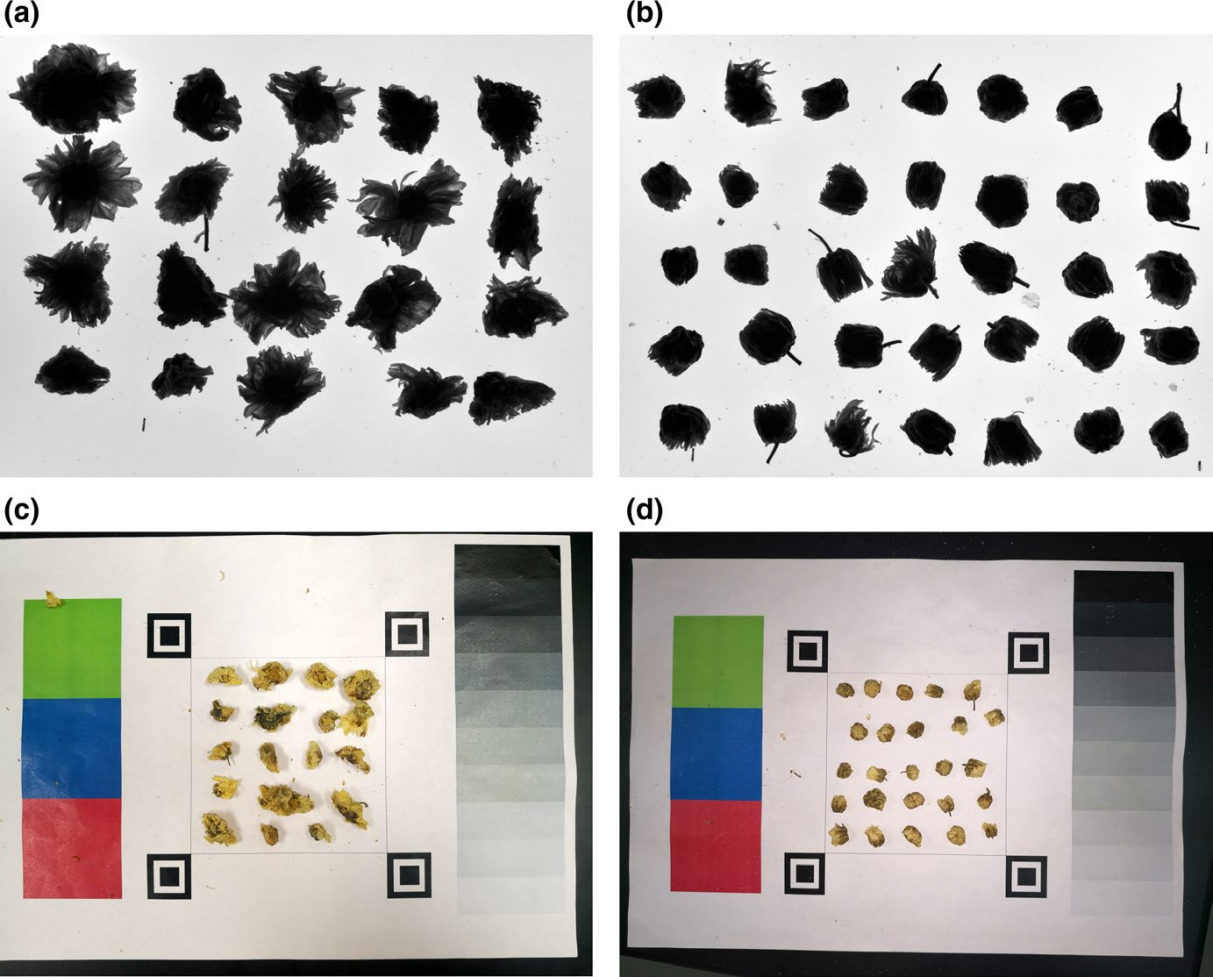
Representative contour images of chrysanthemum teas. (a) Hangbaiju by a gel imager, (b) Hangtaiju by a gel imager, (c) Hangbaiju by a smartphone, and (d) Hangbaiju by a smartphone

The raw images acquired from the gel imager were first visually evaluated. Figure 3 shows typical raw images of HB and HT subjected to CVS processing. Compared with the open‐field or hand‐held smartphone without fixed lighting, the gel imager can acquire better‐quality images with less interference (Figure 3a and b). However, due to the limitation of hardware setup, all the color and texture information were discarded. The reason is that the light source, the sample, and the camera were aligned vertically to ensure the most effective extraction of morphological features (Figure 1). On the other hand, further image analyses of shape factors were greatly simplified.

It would be interesting to characterize the morphological features of fetal and common chrysanthemum teas to further understand the differences of functional component compositions. The morphological features were calculated by the CVS routines and were summarized in Table 3. In general, the fetal chrysanthemum teas had smaller values in many features than the bloom counterparts. These typical features such as perimeter, area, long‐ and short‐axis length, incircle, and excircle length were closely related to sizes. In particular, HG yielded the largest average size features, while the KM and HJ were shortest in perimeter, long‐, and short‐axis lengths, smaller in area than any other teas. The result was consistent with the Chinese Pharmacopoeia (Chinese Pharmacopoeia Committee, 2015), in which HB is around 1.6 times larger in diameter than HJ. The results also agreed with the fact that the teas were steadily grow larger in their late flowering stage. However, there were no clear differences between fetal and bloom chrysanthemum teas. Specifically, the differences of both perimeter and area of KX and HT were closer, though they were collected during different flowering stages. Therefore, precise determination of the flowering stage and types of teas solely on the size parameter alone is not adequate for decision. There are no evident differences between either fetal and bloom or among different types of teas in other features such as aspect ratio, compactness and roundness based on univariate comparisons.

**Table 3:** Summary of morphological features

Features	HB	HG	KX	HT	KM	HJ	DT
Perimeter	715 ± 169	1,112 ± 279	490 ± 116	427 ± 90	254 ± 55	252 ± 76	387 ± 84
Area	19,504 ± 7,433	36,722 ± 13,039	9,601 ± 2,869	9,156 ± 2,571	3,960 ± 1,364	2,914 ± 961	7,850 ± 2,399
Long‐axis length	211 ± 41	284 ± 51	149 ± 33	137 ± 27	82 ± 19	85 ± 28	126 ± 28
Short‐axis length	133 ± 32	190 ± 44	99 ± 19	101 ± 17	68 ± 13	57 ± 11	91 ± 15
Incircle radius	65 ± 14	95 ± 20	48 ± 7	47 ± 7	32 ± 6	27 ± 5	43 ± 6
Excircle radius	108 ± 21	145 ± 27	76 ± 17	70 ± 15	42 ± 10	43 ± 14	64 ± 15
Area equivalent diameter	254 ± 48	349 ± 63	180 ± 35	168 ± 29	105 ± 21	101 ± 27	155 ± 28
Circularity	155 ± 29	213 ± 37	109 ± 16	107 ± 15	70 ± 12	60 ± 10	99 ± 15
Shape parameter	2.16 ± 0.48	2.75 ± 0.65	2.04 ± 0.59	1.62 ± 0.42	1.35 ± 0.38	1.81 ± 0.77	1.56 ± 0.41
Aspect ratio	1.63 ± 0.36	1.54 ± 0.29	1.54 ± 0.35	1.36 ± 0.24	1.23 ± 0.42	1.51 ± 0.57	1.41 ± 0.40
Compactness	0.74 ± 0.07	0.75 ± 0.06	0.75 ± 0.09	0.80 ± 0.09	0.86 ± 0.09	0.75 ± 0.14	0.80 ± 0.10
Roundness	0.61 ± 0.10	0.66 ± 0.10	0.64 ± 0.11	0.69 ± 0.10	0.77 ± 0.11	0.66 ± 0.15	0.69 ± 0.12
Irregularity	259 ± 169	346 ± 211	153 ± 214	81 ± 134	30 ± 107	92 ± 178	81 ± 135
Normalized irregularity	0.04 ± 0.02	0.03 ± 0.02	0.04 ± 0.04	0.02 ± 0.03	0.02 ± 0.04	0.06 ± 0.07	0.03 ± 0.03

For color image subjected to DNN modeling as shown in Figure 3c and d, it is demonstrated that there are visible variations of position, lighting, shadow, and color during image capture due to the random fluctuations caused by the operator and the environment. Such differences brought additional challenge to the correct identification of tea types. Although the CVS is popular for food quality assurance, it is suggested that the CVS hardware, especially lighting may greatly affect the image quality and lead to poor recognition (Jahr, 2006). As DNN is reported to be a robust method, it is interesting to examine the overall performance.

### 3.2 Principal component analysis of chrysanthemum teas by their shape features

**Figure 4:**
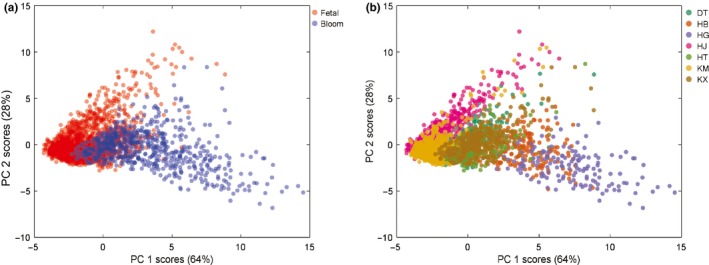
Principal component scores plots of chrysanthemum contour features by flowering stage (a) and tea type (b)

Since the univariate approach was insufficient to determine the type of chrysanthemum teas, a multivariate model may be a suitable alternative. The PCA was performed to provide initial analyses of the multivariate relationships according to the whole contour feature set. Figure 4 is the principal component score plot of chrysanthemum teas based on the contour features. The score coordinates of each sample were marked by both flowering stage upon collection and by product types. The combined explained variance by the first and second largest latent variables (PC 1 and PC 2) were 92%, indicating a minimal PCA model built with the first two largest principal components is already able to effectively explain most part of the multivariate correlations. Therefore, no further observation of smaller principal components was included. No clear outlier is detected from the dataset. It can be observed that the distribution of fetal and common chrysanthemums is significantly different and largely separated, where the fetal and bloom teas located in the upper left and bottom right side (Figure 4a). This result is consistent with the general rule of morphological differences between the fetal and bloom chrysanthemums, where the physical appearances of the fetal chrysanthemum should be larger than the bloom counterparts.

Differentiating seven individual tea types is more challenging since the number of classes is larger than classifying flowering stages, while the number of instances in each class lower. A limited extent of separation between different type of teas can also be observed (Figure 4b). Samples HJ and HG were located on the boundary of data clusters, indicating that they were the most characteristic samples classes for fetal and bloom teas, respectively. However, overlapping occurred in many classes of samples, especially KX and DT spanned also the same region in the scores plot, despite that KX and DT belongs to different flowering stage. The overlapping suggested that classification of individual tea type is more challenging that classification of flowering stage, due that the extent of overlapping between different types of teas. The result indicated PCA is also suitable for discovering the multivariate morphological regularities of teas according to their contour features, while conventional methods including direct comparisons may not achieve. Overall, the PCA did not led a conclusive and clear separation. Therefore, further multivariate modeling techniques were applied to achieve quantitative models.

### 3.3 Classification by shape features of chrysanthemum teas

Since different tea samples overlapped from the PCA scores plots, further multivariate classification is necessary. Besides, the PCA scores plots offered visual interpretation to find relevant patterns between individual contours image; however, they were not appropriate for nonlinear modeling and automated sample classification. The multivariate classification can achieve automatic decision‐making without judging the output, in addition that they were capable of handling nonlinear behavior. The prediction accuracies of the KNN, MLP, and SVM multivariate classifiers were shown in Table 4. All the three classifiers performed well in the classification of flowering stage. The best result was achieved using the SVM, which yielded 90% prediction accuracy for the test set. Although only with a slight performance increment, comparing the different multivariate modeling techniques, SVM can be an adequate for modeling shape features. It can be concluded that computer vision combined with chemometrics can effectively and automatically distinguish chrysanthemums collected at fetal or at blooming stage.

**Table 4:** Best prediction accuracies obtained by different multivariate classification models

	Flowering stage		Tea type	
	Training	Test	Training	Test
KNN	88	87	57	55
MLP	90	88	64	62
SVM	91	90	64	63
DNN	100	96	100	89

Abbreviations: DNN, deep neural network; KNN, k‐nearest neighbor; MLP, multiple linear perceptron; SVM, support vector machine.

When classify tea types, however, the differentiation was only between 55% and 63% on the test set using all three classifiers, indicating that the shape parameter alone is not sufficient in differentiating various commercial tea types. The best classification in the optimal SVM prediction results from the test set occurred among different fetal chrysanthemum teas, where 11 out of 34 HT samples were misclassified as DT, while 12 out of 36 DT samples were misclassified as HT. The misclassifications demonstrated great amounts of shape consistencies in fetal chrysanthemum teas. Further CVS techniques, such as to include texture and color, should be required to achieve a proper model for this specific task.

### 3.4 DNN classification of chrysanthemum tea by raw images

A novel approach using the deep neural network with entire colored image approach was also used and compared with the conventional counterparts by shape factors. One of the most attractive features of DNN is that the images can be directly used as modeling inputs; therefore, minimal data‐preprocessing is required. The network architecture of the DNN was first optimized. A series of networks with different architectures were trained and compared. All candidate structures and their respective performances classifying tea types were given in Table 5. Generally, all models yielded significantly higher accuracies over the test set compared with the previous morphological feature approach, suggesting that the color and texture information may be the important characteristics when visually differentiating chrysanthemum teas. It is generally accepted in deep neural networks that the number of filters and layers should increase when the problem is more complex (Muñoz et al., 2019). However, up to a certain level, the architecture may finally lead to overfitting. Among different DNN models, all the hyperparameters have certain optimal values. Specifically, different network depth ranging from 2 to 5 autoencoders were evaluated, and the proper size of the network is found to be 4. The best pooling layer sizes were 2, 8, 4, and 2. The proper convolution filter size was 2 × 2. The final optimal network architecture for the DNN training was determined to included four autoencoders with consecutively 32, 64, 128, and 256 neurons in the convolutional layer, in which all convolutional filters were with a size of 2 × 2. The DNN model yielded highest prediction accuracies of 96% and 89% when classifying flowering stage and tea type as given in Table 5, respectively, demonstrating that the DNN may be advantageous for automatic visual quality determination. The results that DNN modeling yielded comparable, if not superior performance than the models generated by manually selected imaging features were also consistent with other studies in food quality assessment (Muñoz et al., 2019; Pfisterer et al., 2018; Xu & Sun, 2018). Therefore, the automated and high‐throughput visual authentication and quality assurance of food and may be among one of the potential areas of application of DNN, where higher accuracy may be achieved than the traditional multivariate image classification approaches. Additionally, the recognition was achieved by manually taking images of the samples using a regular mobile smartphone without any external calibration, thus leading to great convenience in large‐scale application.

**Table 5:** Performances of different deep network architectures

Network neuron size^a^	Pooling filter size^a^	Convolution filter size	Flowering stage		Tea type	
			Training accuracy	Test accuracy	Training accuracy	Test accuracy
32, 64, 128, 256, 256	2, 8, 4, 2, 1	2 × 2	100	96	100	87
32, 64, 128, 256	2, 8, 4, 2	2 × 2	100	96	100	89
32, 64, 128, 128	2, 8, 4, 2	2 × 2	100	96	100	84
32, 64, 128	2, 8, 4	2 × 2	100	95	95	85
32, 64	2, 8	2 × 2	99	94	100	85
32, 64, 128, 256	2, 8, 4, 2	3 × 3	100	96	100	85
32, 64, 128, 256	2, 8, 4, 2	4 × 4	97	95	100	89
32, 64, 128, 256	2, 8, 4, 2	5 × 5	100	94	100	82
16, 32, 64, 128, 128	1, 4, 2, 1, 1	2 × 2	100	95	100	87

a The series of numbers indicate sizes of hidden layers from the input layer (left) to the output layer (right).

## 4 CONCLUSION

In this study, the CVS combined with multivariate classification and DNN was examined in the quality detection of chrysanthemum teas without requiring any specific hardware. The result demonstrated that according to the shape factors calculated from the contour image of chrysanthemum teas, the PCA is suitable for the initial discovery of the possible distribution of tea qualities, and multivariate model could effectively and automatically distinguish teas collected at fetal or bloom stage. The DNN outperform other approaches, especially in classifying tea types. Besides, the feature of the DNN that accepts raw images as input without further feature extraction is especially attractive for rapid prototyping.

Although the computer vision is a valuable technique to provide efficient and objective detection for food manufacturing companies, specialized hardware is required to keep the quality of image consistent and reproducible. This probably is the reason why computer vision is also referred to as machine vision. On the contrary, a gel imager or a smartphone as the general‐purpose image acquisition device can overcome the drawback that CVS requires specialized machines built with domain‐specific electronic and mechanical parts. Generally, the more variations in these illumination factors, the poorer the classification performance, or vice versa (Jahr, 2006). The utility of a hand‐held device in this study with ambient lighting inevitably introduced variances in lighting‐related factors, such as the intensity, direction, and daylight temperature, as can be observed from the differences of the raw images taken from the smartphone. However, DNN yielded a robust and consistent performance by 96% and 89% classification accuracies when differentiating flowering stages and tea types, respectively. The results were consistent with previous findings that DNN can be a useful alternative modeling method for computer vision applications in foods (Muñoz et al., 2019; Pfisterer et al., 2018; Xu & Sun, 2018). It showed promising results when the lighting, environmental background, and camera setup greatly affected the image quality. Both approaches in this study demonstrated that a common, commercially available gel imager or a smartphone can be used as rapid alternatives to the specialized imaging device for CVS, which brings convenience when building an initial CVS classification model at the trial phase as the prototype building of specific imaging hardware is avoided. Therefore, the approaches may be suitable in the initial development of a CVS model so that the model of CVS can be built and validated prior to the hardware development.

This study was our preliminary attempt of applying DNN for food quality monitoring; therefore, the sample size was limited. It is expected that more samples could be incrementally included in the model in future applications. The DNN may be suitable for significantly larger amounts of data, which is advantageous in large‐scale manufacturing. This study may promote the modern computer vision technique to be applied in processed food products such as other dried botanicals and roasted nuts. It is also foreseeable that deep learning can be useful in other hyperspectral imaging techniques.

## CONFLICT OF INTEREST

The authors declare no conflict of interest.

## ETHICAL STATEMENT

Neither human nor animal testing were used in this study.
